# Resin tags formation by modified Renewal MI formulations in a carious dentine model

**DOI:** 10.3389/froh.2024.1420541

**Published:** 2024-06-14

**Authors:** Nabih Alkhouri, Wendy Xia, Paul Ashley, Anne Young

**Affiliations:** ^1^ Department of Biomaterials and Tissue Engineering, UCL Eastman Dental Institute, London, United Kingdom; ^2^ Department of Paediatric Dentistry, UCL Eastman Dental Institute, London, United Kingdom

**Keywords:** dental composite, resin tags, caries-like dentine, minimally invasive dentistry, restorative dentistry

## Abstract

**Objectives:**

To determine which components in a new restorative material (Renewal MI) improve its ability to form resin tags within demineralized dentine.

**Methods:**

Varied components included polylysine (PLS), monocalcium phosphate (MCP), powder to liquid ratio (PLR), 4-methacryloyloxyethyl trimellitate anhydride (4META), and polypropylene glycol dimethacrylate (PPGDMA). Urethane dimethacrylate (UDMA), containing PPGDMA (24 wt%) and 4META (3 wt%), was mixed with glass filler with MCP (8 wt%) and PLS (5 wt%). PLR was 3:1 or 5:1. Reducing MCP and/or PLS to 4 and 2 wt% respectively or fully removing MCP, PLS, 4META or PPGDMA gave 16 formulations in total. Renewal MI, Z250 (with or without Scotchbond Universal adhesive) and Activa were used as commercial comparators. Collagen discs were obtained by totally demineralizing 2 mm thick, human, premolar, coronal dentine discs by immersion in formic acid (4M) for 48 h. The restorative materials were then applied on top (*n* = 3), before dissolving the collagen in sodium hypochlorite (15%). SEM/EDX was employed to determine resin tags length, composition, and surface coverage.

**Results:**

Tags were >400, 20 and 200 µm and covered 62, 55 and 39% of the adhesion interface for Renewal MI, Scotchbond and Activa, respectively. With experimental formulations, they were 200 and >400 µm long with high vs. low PLR and composed primarily of polymerized monomers. Percentages of the adhesion interface covered varied between 35 and 84%. Reducing PLS or MCP caused a decline in coverage that was linear with their concentrations. Reducing MCP had lesser effect when PLS or PLR were low. Removal of 4META caused a greater reduction in coverage than PPGDMA removal.

**Conclusion:**

PLS, MCP, 4META, PPGDMA and low PLR together enhance Renewal MI tags formation in, and thereby sealing of, demineralized dentine.

## 1 Introduction

Tooth decay is the most common untreated human disease worldwide. While it affects about 30% of adults, the global, estimated, average prevalence of untreated caries in deciduous teeth is 43%. In 2019, global case numbers amongst children were estimated at about 514 million (1). Additionally, in the UK, tooth extraction due to caries is the most common reason for a young child to require referral to hospital. In England, −24% of 5-year-old children have 3–4 teeth that are decayed or missing but only −10% of these are filled (2, 3). A major cause of non-restoration is difficulties in behavioural management. Additionally, decay can reach the pulp faster in primary compared with larger permanent teeth, at which point extraction may be the only option. Quicker and easier to place restorative materials would help address these issues.

In a carious cavity, the infected dentine immediately below the enamel is generally beyond repair. It can be soft, rich with bacteria and totally demineralized due to mineral solubility in bacterial acids. Any remaining collagen structure may also be collapsed through endogenous enzyme activated hydrolysis. Caries affected dentine closer to the pulp, however, can be repairable. This dentine can be harder, less infected, may have some minerals remaining, and its original collagen tubule structure intact (4–6). In modern dentistry, there is a drive to conserve this affected layer and employ more minimally invasive dentistry (MID). MID reduces the risk of exposing the dental pulp during tooth restoration. It also reduces the need for local anaesthesia and high-speed drilling, which is a major benefit when treating anxious children.

Restorative materials used in MID should ideally seal and halt decay, provide immediate restoration of tooth aesthetic and mechanical properties and encourage underlying, natural tooth repair. Materials employed include glass ionomer cements (GICs) and composite resins (5). The cements are quick and easy to place, due to their ability to bond directly with tooth structures. They are, however, weak and prone to early fracture or loss. Conversely, composite resins are stronger but more difficult to place due to multiple, complex, and time consuming, tooth bonding steps (7). To improve bonding whilst maintaining high mechanical properties, a wide range of easier to place cement/composite hybrid materials have been marketed (8). Bonding to affected dentine, however, can be more difficult than to sound dentine due to enhanced enzyme catalyzed collagen degradation (9).

Matrix metalloproteinase enzymes (MMPs) in dentine and saliva could be responsible for the long-term failure of restorations and the recurrence of disease in dentine. They are deproteinisation agents which can be activated in an acidic environment (created by bacterial acids during carious attack, or even by the acid-etching step prior to bonding procedure). They complete the role of the demineralizing bacterial acids by breaking down the organic content of dentine (collagen) leading to the degradation of the adhesion interface and microleakage (10, 11). Therefore, material that can limit or inhibit this activity would be of great benefit. To achieve that, some researchers suggested adding inhibitors to the bonding systems (such as chlorhexidine, BB94 and GM6001) or crosslinking collagen (12–16). While some results showed that limiting this degradation enhanced bonding strength, the adhesive system used (self-etch or etch-and-rinse) plays a major role. Better results were obtained using non-simplified systems. Most of these studies explained the importance of stabilizing the interfacial, adhesive / dentine hybrid layer for a successful durable restoration. This, however, is more challenging when bonding to caries affected dentine, due to its higher enzymatic activity (4). Therefore, hybrid layer stabilization can become more essential.

To address the above issues, the authors developed Renewal MI. This is a flowable, hybrid (due to use of a range of filler particle sizes) composite. In a First-in-Human clinical safety trial with 7 paediatric patients, Renewal MI was placed without local anaesthetic, drill, or other tooth bonding steps, directly on grossly decayed (ICDAS 5 or 6) primary teeth. These temporary restorations were found to reduce pain whilst the child was awaiting an appointment for tooth extraction under general anaesthesia. Additionally, restorations remained intact upon extraction and subsequent sectioning. Sectioned restorations showed Renewal MI had bonded through interlocking with the decayed, rough dentine structures, and sealed the cavity via extensive tags formation (17). Further laboratory work is required, to understand and potentially improve this effect.

Conventional laboratory testing of dental adhesives involves assessment of sealing ability and bond strengths with sound dentine (18). Renewal MI has demonstrated effective initial sealing of drilled cavities in sound teeth. Contrary to expectations from the above clinical study, however, Renewal MI bond strengths to sound dentine were very low. Conversely, with fully demineralized dentine discs (employed as a new model for affected dentine), Renewal MI penetrated and formed long resin tags, as seen in its clinical trial. Such resin tags would replace water in tubules limiting any enzyme catalysed hydrolysis of dentine. Furthermore, unlike other restorative materials, Renewal MI was proven to inhibit collagenase activity of MMP-1 at the adhesion interface (17, 19).

The aim of the following study was therefore to demonstrate, and explain, which components in Renewal MI encourage tags formation in the above affected dentine model. Renewal MI is a urethane dimethacrylate (UDMA) based composite. Polypropylene glycol dimethacrylate (PPGDMA) is added to the UDMA phase as a diluent monomer to enhance flow and monomer conversion. 4-methacryloyloxyethyl trimellitate anhydride (4META) is included to promote adhesion (20). The glass filler phase (powder) contains particles of monocalcium phosphate (MCP) for remineralization and polylysine (PLS). It is flowable due to a relatively low powder to liquid ratio (PLR, 3:1 by weight), when compared with packable composites. Assuming powder and liquid densities of 3 and 1 g/cm^3^ respectively, this gives a powder volume fraction of 50%.

The null hypotheses are that PLS, MCP and PLR at low vs. high levels have no significant effect on the percentage of demineralized dentine disc / material interface covered by tags or tag length. Furthermore, they have no effect on tag composition, which is also not significantly different from the tag free composite surface. Additionally, complete removal of PLS, MCP, PPGDMA or 4META has no effect on tag coverage at either low or high PLR.

## 2 Materials and methods

### 2.1 Materials

The experimental composite fillers consisted of silane treated, barium aluminosilicate glass with average particle sizes of 7 µm and 0.7 µm (GM27884, Schott, Germany), fumed silica nanoparticles (Aerosil OX 50, Evonik, Germany), PLS (Handary, Belgium) and MCP (Himed, USA). The clear yellow liquid phase contained the acidic, adhesive monomer 4META (Polysciences, USA) and initiator camphorquinone CQ (DMG, Germany). These were dissolved first in the diluent monomer PPGDMA (Polysciences, USA), then a base monomer, UDMA (DMG, Germany). Mixing of powders and liquids was undertaken using a speed mixer (Synergy speed mixer, UK) at 3,500 rpm for 40 s.

Four flowable and four packable composites were initially prepared that had 3 variables (PLR, PLS and MCP) each at 2 levels. Powder to liquid weight ratios (PLR) were 3:1 or 5:1, respectively. The filler phase consisted of aluminosilicate glass of 7 µm and 0.7 µm combined with fumed silica in the weight ratio 6:3:1. PLS (2% or 5%) and MCP (4% or 8%) were added as a weight percentage of the filler. The liquid phase consisted of UDMA (72 wt%), PPGDMA (24 wt%), 4META (3 wt%) and CQ (1 wt%), as in Renewal MI.

Additionally, a further 8 formulations, 4 of each PLR, were then prepared. These had PPGDMA (24 wt%), 4META (3 wt%), PLS (5 wt%) and MCP (8 wt%), but one of each of these components in turn removed. These enable assessment of which components in the liquid phase (PPGDMA or 4META) most affects tags formation and whether effects of PLS and MCP are linear with their concentration. The compositions of all the experimental formulations studied are summarized, and compared with that of Renewal MI (Schottlander, Letchworth, UK), in Table 1.

**Table 1:** Composition of all experimental formulations and renewal MI. CQ was fixed at 1 wt% in the liquid phase. Glass in the filler phase refers to barium aluminosilicate glass of 7 µm and 0.7 µm combined with fumed silica in the weight ratio 6:3:1. All modified Renewal MI formulations were mixed at Powder: Liquid Ratio PLR 3:1 or 5:1. 3 demineralized teeth were restored with each formulation.

Code	Liquid (wt%)			Powder (wt%)			PLR (wt:wt)
	UDMA	PPGDMA	4META	Glass	PLS	MCP	
F1	72	24	3	87	5	8	3:1 or 5:1
F2	72	24	3	91	5	4	3:1 or 5:1
F3	72	24	3	90	2	8	3:1 or 5:1
F4	72	24	3	94	2	4	3:1 or 5:1
F1-PPGDMA	96	0	3	87	5	8	3:1 or 5:1
F1-4META	75	24	0	87	5	8	3:1 or 5:1
F1-PLS	72	24	3	92	0	8	3:1 or 5:1
F1-MCP	72	24	3	95	5	0	3:1 or 5:1
Renewal MI	72	24	3	88	4	8	3:1

UDMA, urethane dimethacrylate; PPGDMA, polypropylene glycol dimethacrylate; 4META, 4-methacryoyloxy trimellitate anhydride; PLS, polylysine; MCP, monocalcium phosphate monohydrates.

Filtek Z250 with and without Scotchbond universal adhesive SBU (3M ESPE, St. Paul, MN, USA) and Activa kids (Pulpdent, Watertown, MA, USA) were used as commercial comparators (Table 2).

**Table 2:** Main components in commercial materials under investigation according to manufacturers.

Material	Type	Manufacturer	Composition	
			Liquid phase	Filler
Filtek Z250 (Shade B3)	Conventional composite	3M ESPE, USA	BISGMA, UDMA, other dimethacrylates	Zirconia/silica (0.6 µm)
Scotchbond universal	Combined Selective Etch, Self-etch, Total Etch	3M ESPE, USA	BISGMA, HEMA, 10 MDP, methacrylate modified polyacid, water, ethanol	Silica
Activa kids (Pedo shade double-barrel syringe)	Bioactive RMGIC	Pulpdent, USA	UDMA, other methacrylates, modified polyacid	Reactive and unreactive fillers, sodium fluoride

RMGIC, resin-modified glass ionomer cement; BISGMA, bisphenol A-glycidyl methacrylate; HEMA, hydroxyethyl methacrylate; 10MDP, 10-methacryloyloxydecyl dihydrogen phosphate.

### 2.2 Demineralized dentine model

**Figure 1:**
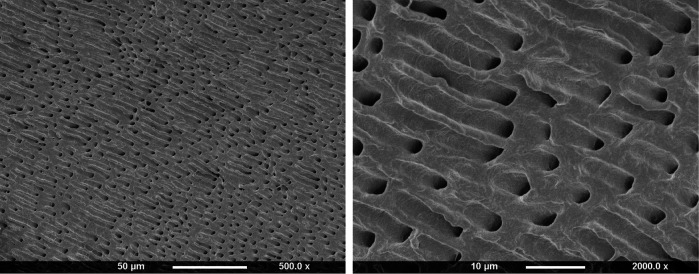
SEM images of 2 mm thick dentine disc after dissolution of minerals in 4M formic acid for 2 days.

Teeth were obtained from the Eastman dental institute biobank, under a generic project ethical approval number 1304 (first dated 29/4/2014 and extended on 13/10/2019, HTA License 12277). Surface occlusal enamel of freshly extracted, sound, human, permanent premolars was first removed using a cutting machine (Leica SP1600, Leica Biosystems, Milton Keynes, UK). Dentine discs of 2 mm thickness were then obtained from the top coronal section. These were immersed in 15 ml formic acid (4M) on a shaker plate for 48 h (Biometra WT16, GmbH, Gottingen, Germany). Gravimetric studies showed mass loss percentage of these discs was proportional to the square root of time until 24 h when it levelled at 75%. This, in combination with EDX (Inca X-Sight 6650 detector, Oxford Instrument, Abingdon, UK) and Raman (Horiba Jobin Yvon, Paris, France) studies of the sample cores, confirmed all minerals had been dissolved. Scanning electron microscopy (SEM, Phillip XL-30, Eindhoven, The Netherlands) showed open dentinal tubules consistent with the collagen physical structure being maintained (Figure 1). Following demineralization, dentine discs were stored in water and used within 1 day.

### 2.3 Tags formation within the demineralized dentine model

Experimental and commercial comparator (Renewal MI, Filtek Z250 with or without adhesive, and Activa) pastes were placed within metal circlips (1 mm thick and 10 mm internal diameter) on top of acetate sheet. Blot dried collagen discs, prepared as above (*n* = 3 per material), were pressed gently on top of the pastes. When Scotchbond universal adhesive was employed, this was applied to the dentine surface for 5 s. It was then air dried and light cured for 20 s, before treated dentine placement on Z250. All materials were light cured for 40 s on each side (Demi Plus, Kerr, Orange, CA, USA, wavelength range: 450–470 nm, intensity from 1,100–1,330 mW/cm). Following subsequent removal from moulds, samples were fully immersed in 10 ml sodium hypochlorite (15%) for 2 days. This totally dissolved any remaining tooth collagen and exposed any tags that had formed at the adhesion interface.

Dentine imprints on the material surfaces were imaged through SEM following sputter coating with Gold/Palladium (under vacuum pressure of 0.5 mbar, current of 20 mA, for 90 s). To determine the composition, lengths, and percentage coverage of resin tags at the dentine/material interface the following studies were undertaken.

Elemental mapping of Renewal MI showed differences in tags free vs. tags covered areas, particularly the levels of carbon, silicon, and oxygen. With the eight F1-F4 formulations, the elemental composition of representative areas on one specimen per formulation was therefore determined using EDX. Where possible, two areas on images at 500× magnification, either covered by tags or tags free, were scanned for each of the eight formulations (F1-F4 of PLR 3:1 or 5:1). Areas examined were approximately 10^4^ µm^2^ but varied in size / shape to ensure they were either rich with tags or completely free of tags. Level of composition variation with changing formulation was much smaller than that observed between tags free and tags rich areas. Additionally, when tags density/coverage was low, obtaining the tags composition (without including the underlying composite) was difficult. Consequently, only the average tags rich and tags free result is provided. Taking the tags free surface carbon content of Renewal MI as 30 +/- 5%, alpha of 0.05 and power at 90%, a group size of 5 would provide a significant effect between two independent groups.

ImageJ v1.8 (NIH, LOCI, University of Wisconsin, Madison, WI, USA) software was employed to randomly measure 10 representative tags where the beginning and tip of the tag could be observed. For longer tags (>400 µm) images at−100× magnification were employed. This was increased up to 800× magnification for shorter tags (−20 µm). With long tags finding both ends could be difficult. In this case only approximate sizes are provided.

Additionally, as areas covered with tags were darker in colour than the tags free areas, percentage of the adhesion interface area covered by tags was obtained by analyzing images of whole specimens with ImageJ. Adhesion areas were typically oval shaped with minimum and maximum diameters of −5 and 7 mm or smaller sections of an oval. Average adhesion area per restoration was 17 (SD = 5) mm^2^. To reduce variability, percentage coverage for each restoration was determined by dividing the tags area by the total adhesion area (excluding the pulp horn region). This reduced the average standard deviation to 6% of the value. Previous studies have shown Renewal MI to have a coverage of 60 +/- 4%. Sample size calculations indicate a reduction with modified formulations to 50% (or increase to 70%), alpha of 0.05 and power at 90%, a group size of 3 would provide a significant effect between two independent groups.

### 2.4 Statistical analysis

All values and error bars reported were the mean with 95% confidence intervals (95% CI). Independent T test in SPSS Statistics v24 for Windows (IBM, Armonk, NY, USA) was used to test significance of different variables on resin tags composition (*p* = 0.05). Univariate Analysis of variance UNIANOVA (or the general linear model) was used to test significant effects of variables on tags composition and coverage area percentages (*p* = 0.01). Levene's test was used to assess homogeneity of variance. As variances were equal, tags coverage data was analyzed using one-way analysis of variance (ANOVA) followed by post-hoc Tukey's test for multiple comparisons (*p* = 0.01) (21). Linear regression of coverage area vs. PLS or MCP wt% in the filler was undertaken using Microsoft Excel v16.64.

## 3 Results

### 3.1 Tags length

**Figure 2:**
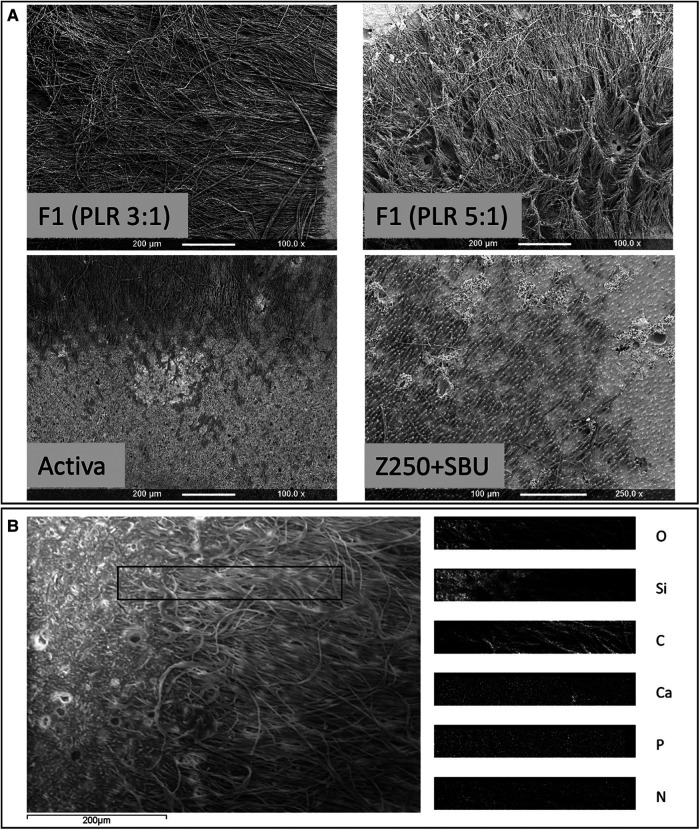
(**A**) Representative SEM images of restorative materials following dissolution of the caries-like collagen to expose the adhesion interface. F1 PLR 3:1 (a-top left corner) or 5:1 (a-top right corner), Activa (a-lower left corner) and Z250 with SBU adhesive (a-lower right corner). Z250 by itself did not show any tags at the interface. Areas rich with resin tags appeared darker in comparison to tag free areas. (**B**) SEM image of Renewal MI surface showing adjacent tags free and tags rich areas. EDX elemental maps of the area in the rectangular box, overlapping both tags and tags free regions, is provided on the right (O, Oxygen; SI, Silica; C, Carbon; Ca, Calcium; P, Phosphorus; N, Nitrogen).

Z250, with no adhesive, gave no tags. Conversely, average tag length of Z250 with SBU adhesive was −20 µm. Activa and all experimental higher powder content (PLR 5:1) composites gave tags of −200 µm. Renewal MI, and experimental formulations with lower powder content (PLR 3:1), however, had tags that were much longer (>400 µm) (Figure 2).

### 3.2 Tags composition (F1-F4 formulations)

**Figure 3:**
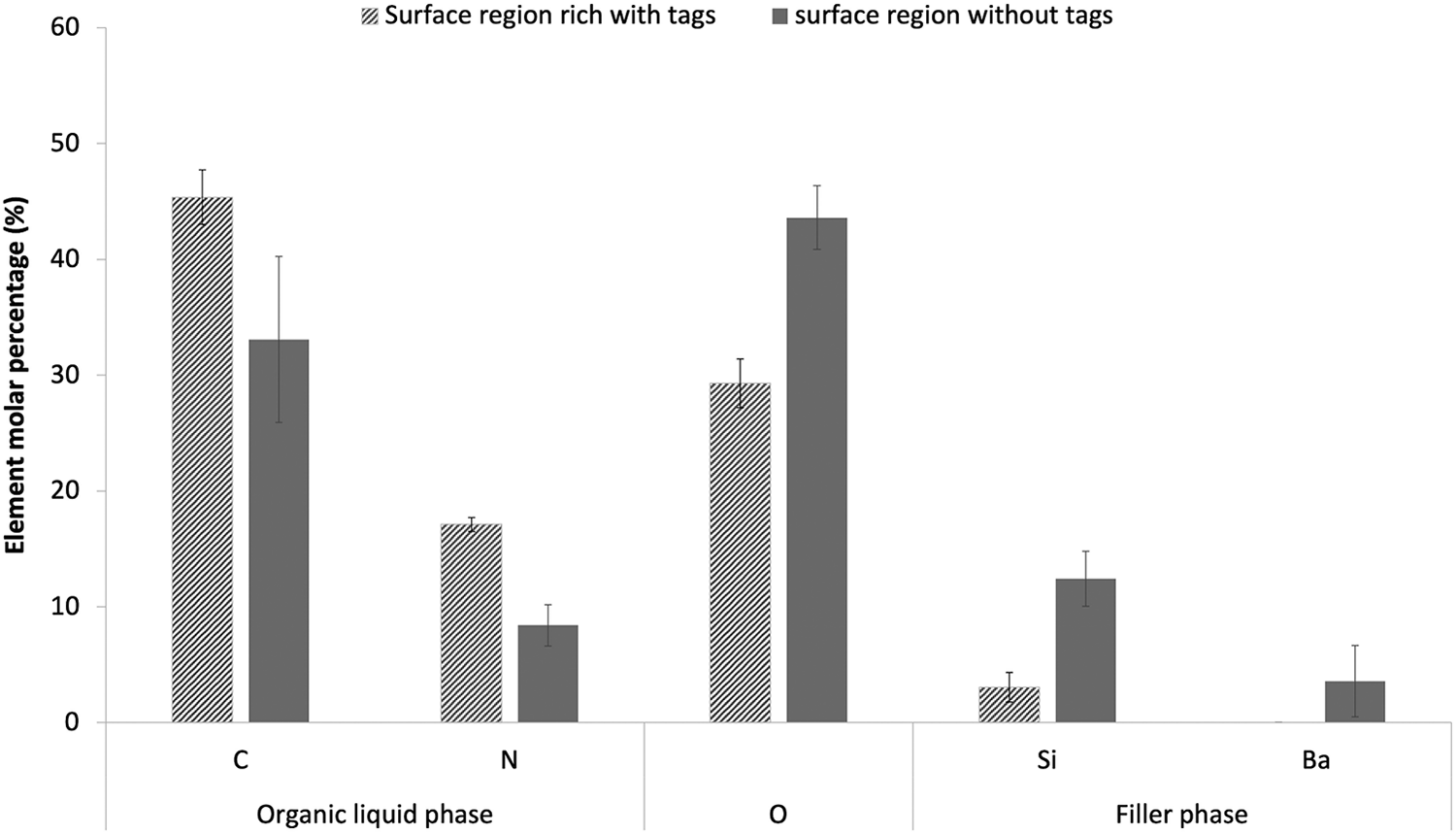
Elemental composition (molar percentage) of adhesion surface regions that were rich versus free of tags. Data are provided as an average result for the eight experimental formulations F1-F4 with PLR 3 and 5. Two areas were examined for each formulation in tags free or tags area. Only elements with >2 molar percentage in either the tags or the body of the composite are shown in the chart. Error bars are 95% CI (*n* = 8) and indicate the variability between formulations. Use of the general linear model indicated no significant effect of material variables. All elements, shown, however, have significantly different percentages in tags compared with tags-free regions**.**

For formulations F1-F4, the general linear model showed that varying PLR, PLS or MCP had no significant effect on the elemental compositions of either tags or tag free areas within the dentine imprint regions (*p* > 0.01). Independent T test, however, showed significant differences in Carbon (C), Nitrogen (N), Oxygen (O), Silicon (Si) and Barium (Ba) in tags rich vs. tag free areas (*p* < 0.05). Average percentages of these elements for the eight F1-F4 formulations are provided in Figure 3. C and N (from organic polymeric matrix phase and polylysine) were observed to be higher in tags rich regions. Oxygen (from fillers and matrix phase) was lower in the tags rich area. Si and Ba (from glass filler particles) were lower and absent, respectively, in the tags. Calcium and phosphorus (main elements in MCP) were generally at too low a level to detect.

### 3.3 Tags coverage area

#### 3.3.1 Commercial and F1-F4 formulations

**Figure 4:**
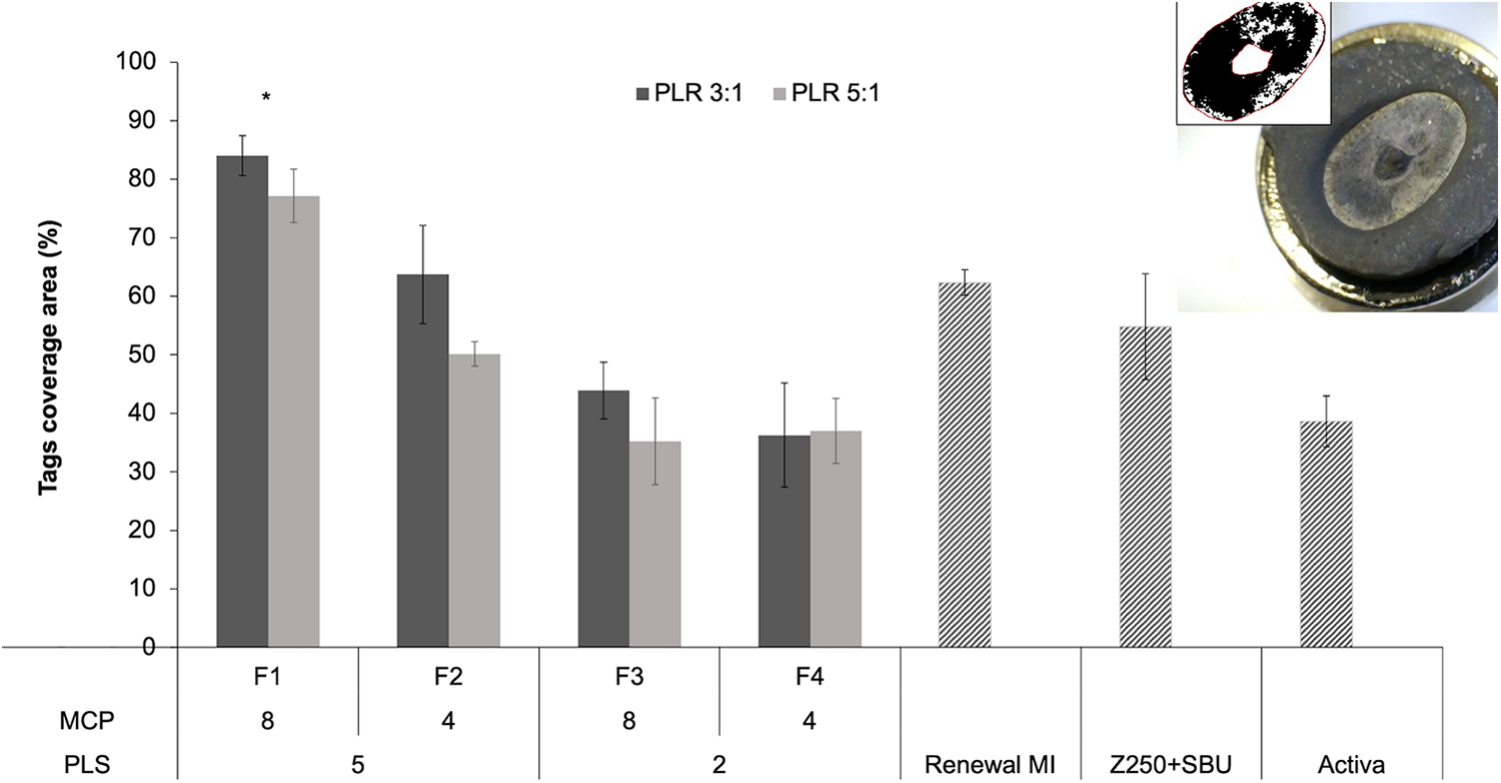
An example lower magnification image of a sputter-coated composite disc following dissolution of collagen is provided in the top right corner. The central raised region is an imprint of the pulp horn space and was ignored in the analysis. Other darker areas raised within the imprint area are rich in resin tags. The black and white image of the collagen imprint area shown was used with ImageJ to quantify areas covered by tags. The bar chart presents tags area percentages for commercial comparators and experimental formulations F1 to F4. Experimental materials had 8 or 4 wt% MCP and 5 or 2 wt% PLS in the powder phase (P). The liquid (L) had 24% PPGDMA and 3% 4META. PLR was high (5:1) or low (3:1). Error bars are 95% CI (*n* = 3). The * indicates that F1 was significantly different from all other groups regardless of PLR. Statistical analysis (One way ANOVA followed by Tukey`s pairwise comparisons) did not show significant differences within each formulation (when other variables were fixed).

Figure 4 shows the area covered by tags at the adhesion interface with formulations F1 to F4 (PLR 3:1 or 5:1), Renewal MI, Z250 (with SBU adhesive) and Activa. The image at the top right corner shows an example of the whole impression area generated after dissolving the demineralized dentine on the surface of a composite. The image is darker in regions rich with resin tags or the pulp horn space in the centre of the imprint area.

Areas of tags coverage, obtained by analyzing these images, were 62, 55% and 39% for Renewal MI, Z250 (with SBU adhesive) and Activa, respectively. For the experimental formulations, highest coverage was observed with F1 (84% and 77% at low vs. high PLR). These have both PLS and MCP at high level. Analysis of F1-F4 (PLR 3:1 or 5:1) data, using a general linear model, showed that the level of variable effect decreased in the order PLS (*p* = 10^−10^) > MCP (*p* = 10^−5^) > PLR (*p* = 0.004). On average, the area covered by resin tags was increased with higher PLS (5 wt%) and MCP (8 wt%) in the powder. There was also, however, a significant interaction effect between MCP and PLS (*p* = 0.0001). Doubling the MCP content significantly increased coverage when the PLS level was 5 wt% but not when PLS was 2 wt%. On average, tags area was increased with lower PLR (3:1) consistent with a beneficial effect of higher liquid content. One way ANOVA and Tukey`s pairwise comparisons showed that F1 (regardless of PLR) had significantly higher tags coverage area in comparison to all other experimental and commercial groups.

#### 3.3.2 Amended F1 formulations

**Figure 5:**
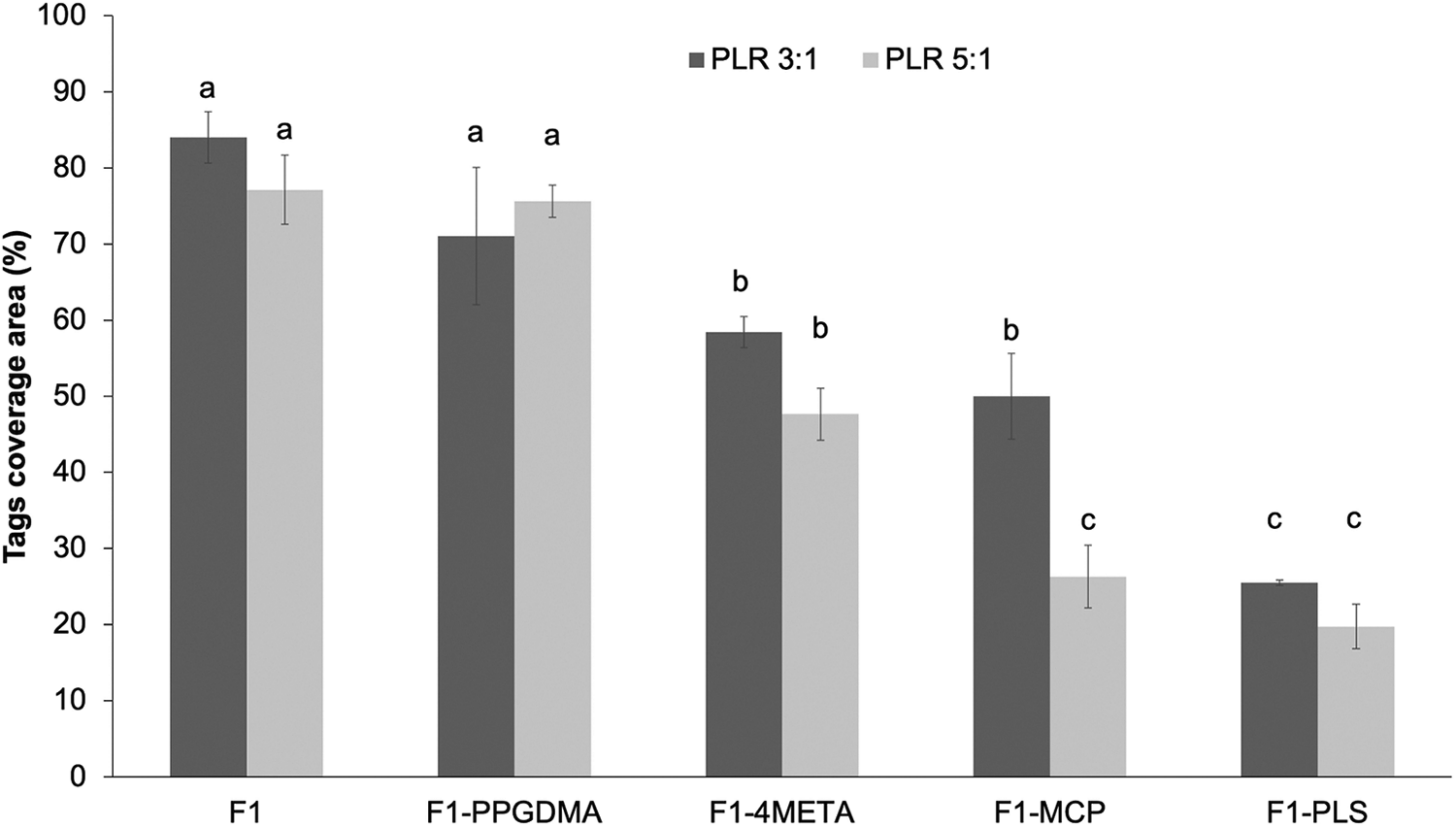
Tags coverage area of F1 in comparison to F1 upon removal of PPGDMA, 4META, MCP or PLS, in turn (all at high versus low PLR). Error bars are 95% CI (*n* = 3). The letters on top of bars represent the groups which are not significantly different from each other (following One-way ANOVA test and Tukey`s pairwise comparisons).

Figure 5 shows that when either PLS, MCP, 4META or PPGDMA were removed from F1, a decrease in tags coverage area was observed. Comparing F1 with each altered formulation in turn (univariate analysis of variance UNIANOVA) showed that the level of variable effect decreased in the order; PLS (*p* = 10^−11^) > MCP (*p* = 10^−8^) > 4META (*p* = 10^−7^) > PPGDMA (*p* = 0.005). Reducing PLR caused a significant increase in coverage area upon MCP removal (*p* = 10^−5^). This PLR effect was not significant upon removal of PPGDMA, 4META or PLS or in the original F1 formulation (*p* > 0.01). One way ANOVA also showed that removing PPGDMA did not result in significant decrease while removing 4META, MCP or PLS did (*p* < 0.01).

Combining data for F1 (5 wt% PLS) and F1-PLS (0 wt% PLS) with results for F3 (2 wt% PLS) above shows that, with all variables other than PLS fixed at their high values, coverage increased linearly with PLS level. Linear regression gave the same gradient of 11.8 +/-0.1% per wt% PLS (R^2^ >0.97) irrespective of PLR. Conversely, combining F1 (8 wt% MCP) and F1-MCP (0 wt% MCP) data with those of F2 (4 wt% MCP) above showed that coverage increased linearly with MCP level but was PLR dependent. In this case, coverage increased by 6.4 and 4.3% per wt% MCP (R^2^ >0.98 in both cases) with high vs. low PLR.

## 4 Discussion

The null hypotheses that PLS, MCP and PLR do not affect experimental composite tags composition could not be rejected. The tags did, however, have significantly different elemental composition from that of tags-free areas. Additionally, the tag lengths were clearly increased by lower PLR. It was also possible to reject the null hypothesis that PLS, MCP, 4META, PPGDMA and PLR have no effect on the percentages of the adhesion interface covered by tags.

Previous attempts to create a caries-like model have used bacteria, acids, or buffer solutions (22–24). Formic acid, however, has been the primary laboratory method that can readily demineralize further than 500 µm without loss of tubule structure (22, 23, 25). Formic acid is known to be one of the highest cariogenic acids produced by bacteria in dental plaque (26). The efficacy of formic acid in creating an affected dentine-like model was consistent with other studies and may be due to the high solubility of calcium formate (27, 28). In this study, the loss of the Raman apatite peak at 960 cm^−1^, disappearance of Ca and P ions in EDX and gravimetric studies (−70% mass loss) were all consistent with total demineralization throughout the 2 mm thick dentine discs (17).

While resin tags formation has been studied extensively in the literature, it is widely thought it does not play a major role in adhesion to tooth structure and restoration durability (29, 30). However, the longer the resin tags, the more stable the adhesion interface should be, especially with the presence of complete hybridization of collagen fibers (31, 32). This study has only looked at the chemical variables affecting resin penetration in Renewal MI, but more work is needed to test the hybrid layer, lateral branches into inter- and intrafibrillar space and the stability of the adhesion interface over time.

For resin tags to form in demineralized dentine tubules, water needs to be replaced. Tags could potentially improve interlocking between dentine and the restoration. Water replacement by tags could also reduce enzyme catalyzed collagen hydrolysis, bacterial nutrient ingress, and space for residual bacterial growth (19).

The above studies suggested that hydrophilic components in the experimental composites (PLS and MCP) increase surface coverage by resin tags. The primary factor affecting tags length, however, was the PLR. The formulations with PLR 3:1 contain 25 wt% liquid compared to 17 wt% in the formulations with PLR of 5:1. The excess monomer appears to be responsible for enabling longer resin tags. Longer resin tags with the more flowable lower powder content experimental formulations (PLR 3:1) are consistent with other studies with flowable composites (33, 34). Lack of Ba and reduced Si content in the tags-rich areas combined with higher C and N suggest that only fluid monomer molecules and to a lesser extent silica nanoparticles, are sufficiently small to diffuse far into the tubules. Larger Ba-containing aluminosilicate glass, PLS and MCP particles may be unable to diffuse far in the short time between material placement and cure. Shorter tags with high PLR may be due to less monomer being available to phase separate.

Activa had comparable tags length to the high powder content (PLR 5:1) experimental formulations. This was despite Activa having overall, a lower viscosity. This might be due to dissolved polymers in Activa causing the separate liquid phase to be more viscous and less able to diffuse. Furthermore, patchy coverage with Activa suggests there may be pools of water on the dentine surface that the material is unable to absorb. These could block Activa penetration into the tubules. A recent study showed that Activa has suffered from high volumetric contraction, poor marginal adaptation and therefore high microleakage with time (35). This might explain the high annual failure rate (24%) of Activa in a one-year clinical trial (36).

Z250 has minimal excess monomer that could phase separate and no hydrophilic components to absorb surface water. The short length of tags obtained with SBU may be due to the low amount of adhesive placed on the dentine surface.

The most important factor increasing coverage area with the experimental materials, was the concentration of PLS. A linear dependence on PLS concentration, within the range of levels tested, was found. When the experimental formulations were applied to demineralized dentine, the resin may be drawn into the tubules by attraction to the hydrophobic collagen and capillary action. This would result in water being expelled into the adhesion surface limiting wider tags coverage. The hydrophilic PLS particles are too large (−20 μm) to penetrate tubules but may absorb this surface water.

The second most important factor affecting coverage was MCP. Increasing MCP, however, was of particular benefit when the powder content was high. Furthermore, the results suggest that MCP and 4META are both beneficial primarily when PLS is present at higher level. A possible explanation of the variable interaction effects is that MCP and 4META react competitively with water absorbed by the PLS. This would thus enable further water sorption. MCP reaction with absorbed water could result in production of dicalcium phosphate dihydrate (brushite) crystals and phosphoric acid. Water with 4META produces 4MET with 2 carboxylic acid groups replacing the anhydride (17, 37–40). These competitive reactions might explain why, when MCP is removed, a higher liquid content (that contains 4META) may compensate for its removal. The positive effect of reducing PLR on tags coverage is explained by higher flowability and more free monomers to separate as discussed previously. PPGDMA beneficial effect may be due to it improving monomer flow. Its effect, however, is small in comparison to increasing PLS, MCP, 4META or liquid content.

Limitations of the study include that, whilst the carious dentine model provides reproducible tags coverage areas, in patients, carious dentine can have varying levels of demineralisation, degradation, bacteria, and enzyme activity. These could all have differing effects on the ability of a material to penetrate and seal the tooth structure. Conventional bond strength determination with fully demineralized dentine is difficult due to its low stiffness. Further work, however, should be undertaken to assess bond strengths vs. level of dentine demineralized. Additionally, other properties such as paste shelf-life, material strength and wear resistance vs. time, water sorption and solubility need to be known to enable selection of a material suitable for clinical trial.

## 5 Conclusions

Polylysine is crucial for ensuring higher percentage of resin coverage area whilst low powder content (PLR 3:1) enabled much longer resin tags in demineralized dentine. MCP and 4META can increase the benefit of PLS possibly through reacting with the water that it absorbs. Reducing monomer viscosity through PPGDMA addition provides only minor benefit. Therefore, formulation F1 provided the highest tags coverage which was significantly greater than that observed in Renewal MI. It's coverage is close to the maximum possible of 100%. Extensive tags formation could help explain the First-in-Human clinical trial success of Renewal MI when used with minimally invasive dentistry.

## Data Availability

The raw data supporting the conclusions of this article will be made available by the authors, without undue reservation.
